# Changes in the gut microbiota during Asian particolored bat (*Vespertilio sinensis*) development

**DOI:** 10.7717/peerj.9003

**Published:** 2020-05-12

**Authors:** Zhongwei Yin, Keping Sun, Aoqiang Li, Deyi Sun, Zhongle Li, Guohong Xiao, Jiang Feng

**Affiliations:** 1 College of Life Science, Jilin Agricultural University, Changchun, China; 2 Jilin Provincial Key Laboratory of Animal Resource Conservation and Utilization, Northeast Normal University, Changchun, China; 3 College of Animal Science and Technology, Jilin Agricultural University, Changchun, China

**Keywords:** Vespertilio sinensis, 16s Amplicon sequencing, Ontogeny, Gut microbiota

## Abstract

**Background:**

The gut microbiota is closely linked to host development, diet and health and is influenced by both the host and the environment. Although many studies have focused on the dynamics of the gut microbiota during development in captive animals, few studies have focused on the dynamics of the gut microbiota during development in wild animals, especially for the order Chiroptera.

**Methods:**

In this study, we characterized the gut microbiota of the wild Asian particolored bat (*Vespertilio sinensis*) from 1 day to 6 weeks after birth. We explored the changes in their gut microbial community compositions, examined possible influencing factors, and predicted the feeding transition period.

**Results:**

The gut microbiota changed during the development of *V. sinensis*. The alpha diversity of the bats’ gut microbiota gradually increased but did not change significantly from the 1st day to the 4th week after birth; however, the alpha diversity decreased significantly in week 5, then stabilized. The beta diversity differed slightly in weeks 4–6. In week 4, the fecal samples showed the highest diversity in bacterial community composition. Thus, we predicted that the potential feeding transition period for *V. sinensis* may occur during week 4. Redundancy analysis showed that age and body mass index significantly affected the compositional changes of the gut microbiota in Asian particolored bats.

**Conclusion:**

The gut microbiota changed during the development of *V. sinensis*. We suggest that changes in the alpha and beta diversity during week 4 after birth indicate a potential feeding transition, highlighting the importance of diet in the gut microbiota during the development of *V. sinensis*.

## Introduction

The gut microbiota plays important roles in the host’s ontogenetic development and helps the host perform a range of physiological activities (McFall-Ngai et al., 2013). The gut microbiota directly affects host health by absorbing energy from food sources (López-Otín et al., 2013) and affects nutrient digestion and absorption in the host’s intestines (Biagi et al., 2016; Flint et al., 2012; Gilbert et al., 2016; Hughes et al., 2018; O’Hara & Shanahan, 2006).

The gut microbial community structure changes as animals grow and develop. Previous studies showed that relatively simple microbial communities colonize the vertebrate gut during animal birth or hatching, and the community develops as the microbial diversity increases during host development (Koenig et al., 2011; Yatsunenko et al., 2012). However, some studies have suggested that maternal vaginal and fecal microbiomes are often the most important inoculation sources in the early stages of life (Gueimonde et al., 2006; Vaishampayan et al., 2010). During vertebrate development, many factors, including dietary, developmental, hormonal and environmental factors, gradually influence host-related changes in the gut microbiota; thus, the microbiota gradually develops into a complex ecosystem (Bataille et al., 2018; Bevins & Salzman, 2011; Brown & Cai, 2007; Conlon, 2011; David et al., 2013; Davis, Bigler & Woodhams, 2017; Longo et al., 2015; Schreiber & Brown, 2003).

Recent studies have examined the composition and function of the gut microbiota during development in mice, pigs, fish and birds. Most studies focused on commercial animals that are food sources for humans. For commercial fish, the gut microbiota diversity increases significantly as the fish develops and diversity stabilizes after the feeding stabilizes and the individual matures (Bledsoe et al., 2016; Li et al., 2017). A longitudinal investigation of age-related bacterial diversity in commercial pig feces yielded similar results (Kim et al., 2011). A study on young pigs found no obvious changes in gut microbiota diversity; however, the diversity differed significantly before and after a feeding transition, then stabilized after weaning (Frese et al., 2015).

However, limited studies are available on gut microbial communities during the development of wild animals. The gut microbial communities of adult wild house sparrows (*Passer domesticus*) differed significantly from those of young wild house sparrows but did not change significantly during the young birds’ development (Kohl et al., 2019). This is similar to changes in the gut microbiota of barn swallows (*Hirundo rustica*) (Kreisinger et al., 2017). However, great tits (*Parus major*) differed in their gut microbial communities and diversity during development (Teyssier et al., 2018). Thus, during development in wild animals, variations in the gut microbiota may differ.

Bats (order Chiroptera) are nocturnal mammals that can fly and capture prey in complete darkness. Owing to their complex and diverse feeding habits and wide ecological distribution, bats are excellent subjects for studying the gut microbiota. However, information on the composition and structure of the bats’ gut microbial communities is scarce and includes only a few studies on the composition and influencing factors of the gut microbiota in Phyllostomid bats (Carrillo-Araujo et al., 2015; Phillips et al., 2012), isolation and identification of the gut microbiota in short-nosed fruit bats (*Cynopterus brachyotis*) (Daniel et al., 2013), and seasonal changes in the gut microbiota (Xiao et al., 2019a) and structural convergence of the gut microbial community in bats under indoor feeding conditions (Xiao et al., 2019b). Only one study has been published on the gut microbiota during bat ontogeny. That study showed that changes in the gut microbiota of *Myotis myotis* were not significantly influenced by age, likely because of growth requirements and that the gut microbiota stability is conducive to bats maintaining energy during flight (Hughes et al., 2018).

To further understand and supplement the literature on the composition and changes in gut microbial community structure during bat development, we focused on the Asian particolored bat (*V. sinensis*), which is the most common bat species in northeast China. For this species, the parturient period is from June to July, and most females give birth to twins. Jin et al. (2012) conducted relevant studies on the ontogeny of Asian particolored bats and provided an age estimation equation, which provides a good research foundation for inferring the ages of young bats based on morphological data. In our study, to avoid injuring bats and to reflect the changes in their feeding habits, we used fecal samples from the bats to study the composition and changes in their gut microbiota. In addition, fecal samples retain more food-related signals from the hosts than do gut samples (Ingala et al., 2018). Using 16S rRNA gene amplicon high-throughput sequencing, we explored the changes in the gut microbiota structure during the ontogeny of Asian particolored bats and inferred the possible relationship between the microbiota and changes in feeding habits (from milk feeding as infants to insectivores as adults). We further determined the potential factors that influence the composition and dynamics of the gut microbial community.

## Materials and Methods

### Sample collection

From June to August 2018, bats of different ages were randomly collected in the Asian particolored bat habitat under the overpass of Acheng district, Harbin City, Heilongjiang Province, China (E 126.9420, N 45.5519). All bats were identified based on morphological characteristics (Wang et al., 2009). Owing to the height of the overpass (ca. 7–8 m), we used a crane to reach the habitat and gently caught the bats by hand with sterile gloves. Each captured individual was put into sterilized kraft bags to collect their excrement. All sampled individuals were healthy, and their feces was collected using nondestructive methods. After defecation, fecal samples were immediately put into a cryopreservation tube containing RNA preservation solution (TIANGEN Biotech, Beijing, China) using sterilizing tweezers and stored at −20 °C for subsequent sequencing. After collecting the samples, the body surface temperature of each bat was recorded using a Fluke 62 MAX IR thermometer (Fluke, Everette, WA, USA), then the weight was measured using an electronic balance (accurate to 0.01 g), and the forearm length and epiphyseal distance were measured using a digital display caliper (accurate to 0.01 mm) to estimate individual age (Table S1). After the measurements, the bats were immediately released back into the habitat. A total of 45 samples were collected from bats that ranged in age from 1 day to 6 weeks (Table 1). According to the regulations of Wildlife Conservation of the People’s Republic of China (Chairman Decree (2016) No. 47), permits are required only for species included in the list of state-protected and region-protected wildlife species. *V. sinensis* is not an endangered or region-protected animal species, so no specific permission was required. All the studies have been approved by Laboratory Animal Welfare and Ethics Committee of Jilin Agricultural University.

**Table 1:** The division of sample age stage and bats’ morphological parameters and sampling number. Ages were estimated using the age equations of Jin et al. (2012).

Age	Forearm length (mm)	Length of total gap (mm)	Definition of age period	Number of individuals
ca. day 1	15.69 ± 0.29	NA	Day 1	4
ca. days 6–9	23.30 ± 1.34	NA	Week 1	6
ca. days 13–15	30.57 ± 0.87	NA	Week 2	7
ca. days 19–22	30.68 ± 0.90	NA	Week 3	7
ca. day 28	47.73 ± 0.43	NA	Week 4	8
ca. days 35–38	NA	1.91 ± 0.37	Week 5	6
ca. days 41–44	NA	0.91 ± 0.26	Week 6	7

**Note:**

NA, the data were not used to assess individual bat age at that division. Length values shown are means ± SE.

### Age estimation and grouping

According to the age equation of *V. sinensis* estimated by Jin et al. (2012), individual age was highly correlated with body weight and forearm length before 28 days (1) and correlated with epiphyseal gap height after 28 days (2). The estimated age equations (Jin et al., 2012) were as follows:

(1) $${\rm Age} = 0.85 \times {\rm Forearm\ length} - 12.47\ (R^2 = 0.98, P\,\lt\,0.01)$$

(2) $${\rm Age} = -5.02 \times {\rm Length\ of\ total\ gap} + 47.2\ (R^2 = 0.91, P\,\lt\,0.01)$$

We used these age equations to evaluate the possible age of the bats from each bat’s forearm length and epiphyseal spacing. We categorized the ages as follows: day 1, week 1, week 2, week 3, week 4, week 5 and week 6 (Table 1). “Day 1” refers to newborn bats and was determined using the age estimation equation and existence of an umbilical cord.

### DNA extraction

An E.Z.N.ATM Mag-Bind Soil DNA Kit (OMEGA, Norcross, GA, USA) was used to extract total bacterial genomic DNA samples following the manufacturer’s instructions. The extracted DNA was stored at −20 °C for further analysis. For the DNA extraction, a combination of mechanical disruption and chemical methods was used to break up the cells. Mechanical treatment facilitates the release of microorganisms in the samples, and chemical methods facilitate full cell lysis.

### 16S rRNA gene amplicon sequencing

A Qubit 3.0 DNA detection kit (Life Technologies, Carlsbad, MA, USA) was used to precisely quantify the genomic DNA to determine the amount of DNA to add to the PCR reaction. PCR amplification of the V3–V4 region of the bacterial 16S rRNA gene amplicons was performed using the forward primer 341F and the reverse primer 805R (Eiler, Heinrich & Bertilsson, 2012). The PCR consisted of 15 μl 2×Taq master mix (Vazyme, Nanjing, China), one μl primer F (10 μM), one μl primer R (10 μM), 10–20 ng genomic DNA, and 30 μl H_2_O. The thermal PCR cycle included initial denaturation for 3 min at 94 °C, followed by five cycles of denaturation for 30 s at 94 °C, annealing for 20 s at 45 °C, and extension for 30 s at 65 °C. Another 20 cycles were performed, including denaturation for 20 s at 94 °C, annealing for 20 s at 55 °C, and extension for 30 s at 72 °C, with a final extension step for 5 min at 72 °C. Illumina bridge PCR-compatible primers were used for the second round of amplification. The PCR system consisted of 15 μl 2 × Taq master mix, one μl Primer F (10 μM), one μl Primer R (10 μM), 20 ng PCR products and 30 μl H_2_O. The thermal cycle consisted of initial denaturation for 3 min at 95 °C, followed by five cycles of denaturation for 20 s at 94 °C, annealing for 20 s at 55 °C, and extension for 30 s at 72 °C, with a final extension step for 5 min at 72 °C. The PCR products were detected by agarose electrophoresis after PCR. MagicPure Size Selection DNA Beads (TRANSGEN, Beijing, China) were used to purify the PCR amplicons. A Qubit 3.0 DNA detection kit was used to accurately quantify recovered DNA for sequencing after equal mixing at a 1:1 ratio. When equal amounts were mixed, 10 ng of DNA was taken from each sample, and the final sequencing concentration was 20 pmol. Barcoded composite PCR products were sent to the Sangon Biotech Co., Ltd. (Shanghai, China) for Illumina Miseq (2 × 300 bp) sequencing. All raw sequences were deposited into the NCBI Sequence Read Archive under accession numbers SRR9695884–SRR9695940.

### Statistical analysis

First, we removed singletons and very rare sequences and performed quality control of the raw sequencing reads using Prinseq v0.20.4 (Faber-Hammond & Brown, 2016). Second, we used PEAR software to merge the paired-end reads with a maximum of 10% mismatches in the overlap region (Zhang et al., 2013). The sample data were identified and differentiated according to barcode label sequence to obtain the data for each sample (Chen & Jiang, 2014; Gill et al., 2006). We then removed the nonamplified region sequence and corrected the sequencing errors using USEARCH v8.1.1831 (Edgar, 2013) and identified and deleted the chimeric sequences using UCHIME v4.2 with the default parameters (Penton et al., 2013). Moreover, non-bacterial sequences stemming from chloroplasts and mitochondria were deleted by QIIME (1.8.0). Subsequently, we performed BLASTN comparison for the chimeric deletion sequences and representative database sequences and removed the sequences with similarities of >80%. The remaining high-quality sequences were clustered into operational taxonomic units (OTUs) with 97% sequence identity using UCLUST (Edgar, 2010). Taxonomy was assigned using the Ribosomal Database Project classifier (Wang et al., 2007); representative sequences were aligned to the Greengenes database (Caporaso et al., 2010), and a phylogenetic tree was constructed using FastTree2 (Price, Dehal & Arkin, 2010). The OTU table for each dataset was filtered using a minimum cluster size of 0.001% of the total reads (Bokulich et al., 2013).

Using QIIME software (Caporaso et al., 2010), the Faith’s Phylogenetic Diversity (PD), Shannon–Wiener, Chao1, and Simpson indices were used to evaluate the alpha diversity of each sample. Using R, the Shapiro–Wilks and Levene tests were performed to evaluate whether the data presented a normal distribution and homogeneity of variance, respectively. The Kruskal–Wallis test (for the non-normal data distribution) and one-way analysis of variance (ANOVA; for the normally distributed data) were used to evaluate the differences in alpha diversity between samples, and all *p*-values were corrected via the False Discovery Rate.

The changes in beta diversity among the gut microbial community were calculated using the unweighted UniFrac distances, which were computed on rarefied data between the samples. UniFrac distances were performed with the *phyloseq* package in R software (McMurdie & Holmes, 2013). All beta diversity results were visualized using principal coordinates analysis (Gower, 1966). We used PERMANOVA to statistically analyze the beta diversity (Oksanen et al., 2008).

At the phylum level, the histogram showed the gut microbiota compositions at different stages of ontogenesis. The total OTU number of the samples was screened (OTU > 0.1%), and a heat map of the screened data was constructed at the genus level to analyze the changes in microbial community compositions in the bats in the different age groups using the *pheatmap* package in R (Kolde, 2015).

Redundancy analysis (RDA) was performed using Canoco 5 (Ter Braak, 1990) to evaluate whether the factors significantly influenced the bats’ gut microbial communities. RDA analysis results were corrected via Bonferroni correction. We also used the *RNCEP* package (Kemp et al., 2012) in R to download the environmental temperature on each sampling date as an environmental factor, which was used in the RDA analysis.

To determine the functional changes of the gut microbiota before and after the predicted dietary transition, functional characteristics of the bacterial communities were analyzed using PICRUSt (Langille et al., 2013). We determined the closed-reference 97% OTUs and selected the Greengenes database, then used the online Galaxy platform1 to normalize the copy number of each OTU. Each sample’s metagenomic and functional predictions were categorized into Kyoto Encyclopedia of Genes and Genomes (KEGG) pathways, representing the gene count of each predicted metagenome (Kanehisa & Goto, 2000). Functional composition was analyzed using STAMP software (Parks et al., 2014) by comparing mean function abundances via Welch’s *t*-test with a Benjamini–Hochberg correction.

## Results

In total, 2,392,734 original obtained were sequenced from 45 fecal samples and 2,327,983 sequences were retained after quality inspection, with an average effective sequence of 51,733 per sample.

**Figure 1:**
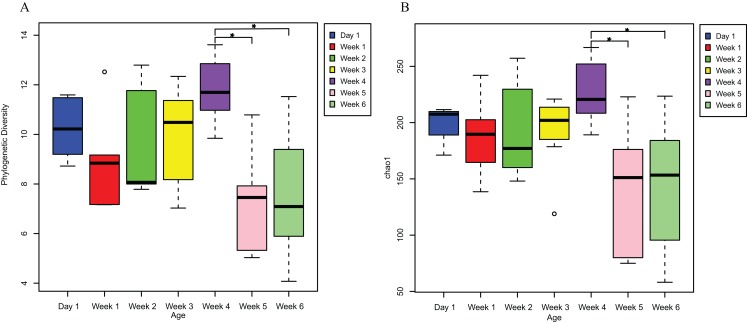
The alpha diversities in Asian particolored bat feces at different ages. This figure represents alpha diversities of feces from Asian particolored bats at different ages. (A) Phylogenetic diversity and (B) Chao1. Asterisks indicate significant differences, *p* < 0.05.

PD analysis showed that the alpha diversity of the gut microbiota of *V. sinensis* did not change significantly from day 1 to week 4 (*p* > 0.05). The diversity for day 1 (mean PD = 10.296) was slightly higher than that of weeks 1 and 2 (Fig. 1). The alpha diversity (PD) increased from week 1 to week 4 (*R*^2^= 0.2285, *p* = 0.01), then decreased significantly at weeks 5 and 6 (one-way ANOVA; week 4-week 5: *p*_adj_ = 0.004; week 4-week 6: *p*_adj_ = 0.005, Fig. 1A). The diversity remained stable in weeks 5 and 6 (Fig. 1A). The Chao1 index showed similar results to those of the PD; the alpha diversity was also positively correlated with age from week 1 to week 4 (*R*^2^= 0.1388, *p* = 0.05), then decreased significantly at weeks 5 and 6 (one-way ANOVA; week 4–week 5: *p*_adj_ = 0.012; week 4–week 6: *p*_adj_ = 0.007; Fig. 1B). The Shannon (Kruskal–Wallis test; *p*_adj_ = 0.5755) and Simpson (Kruskal–Wallis test; *p*_adj_ = 0.612) indices of the gut microbiota communities did not significantly differ throughout the entire *V. sinensis* development.

**Figure 2:**
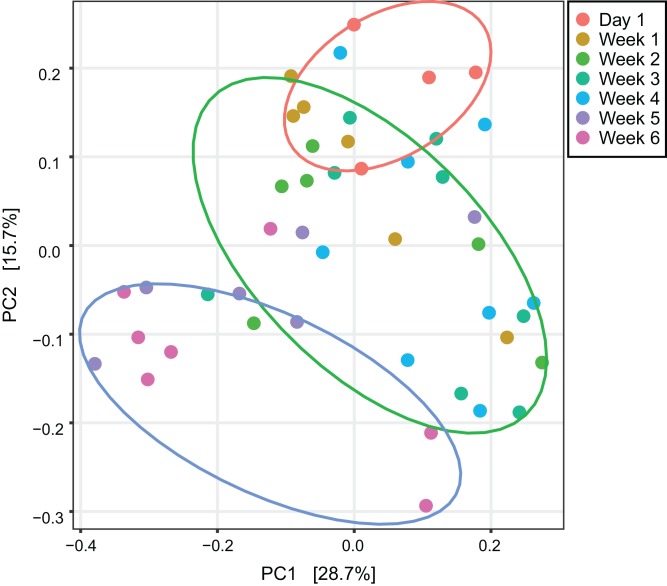
Unweighted principal component analysis. This figure represents unweighted principal component analysis of intestinal microbial diversity of Asian particolored bats at different ages. Ellipses show 95% confidence intervals for three sample types (day 1, weeks 1–4 and weeks 5 and 6).

An unweighted principal component analysis revealed that the beta diversity of the gut microbial communities changed throughout the entire development of *V. sinensis* (PERMANOVA; *p*_adj_ = 0.001, Fig. 2). Most individuals from week 1 and week 4 clustered together, which differed from those in weeks 5 and 6. Day 1 clustering differed slightly from that of the other periods.

**Figure 3:**
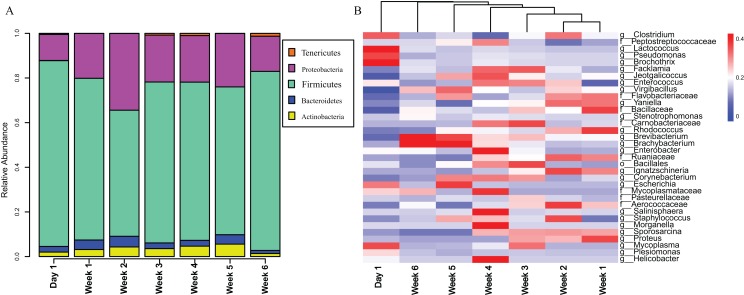
Stacked bar plots and heat map. This figure represents schematic diagram of the bacterial community analysis by age. (A) Stacked bar plots showing the average percentages of bacterial populations at the phylum level in Asian particolored bat feces at different developmental stages. (B) Heatmap showing relative abundances (OTU relative abundance >0.1%) of gut microbiota in Asian particolored bats at the genus level at different developmental stages.

The gut microbiota in *V. sinensis* were relatively stable at the phylum level during ontogeny, with Firmicutes and Proteobacteria constituting most of the community (Fig. 3A). Heatmap analysis at the genus level (OTU relative abundance >0.1%) showed relatively similar bacteria with high abundances from week 1 to week 4 and similar bacteria with relatively low abundances between weeks 5 and 6 (Fig. 3B). Unexpectedly, the community composition on day 1 differed from that during the other periods with high abundances of *Lactococcus*, *Pseudomonas*, and *Brochothrix*. However, the high-abundance bacteria in the other periods were relatively rare on day 1 (Fig. 3B).

Based on the significant changes in gut bacterial diversities between weeks 4 and 5, we divided the experimental groups into week 4-before (including week 4) and week 4-later and performed subsequent functional analyses. However, the results showed that all functions of the gut microbiota before and after week 4 had no significant difference at level 2 and level 3 after Benjamini–Hochberg correction (all *p*_adj_ > 0.05).

**Figure 4:**
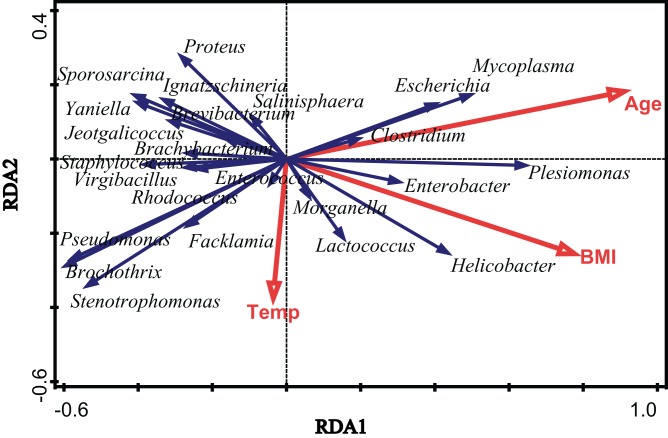
The effects of environmental temperature, age and body mass index. This figure represents the effects of environmental temperature (Temp), age (Age) and body mass index (BMI) on intestinal microbial communities in Asian particolored bats.

To assess the extent of the variation in the microbial community composition that could be explained by environmental factors, we performed an RDA using different categories of environmental factors as explanatory variables. Figure 4 shows the results of a significant RDA model. All factors could account for 19.6% of the variation in the bacterial community structure. Age and body mass index (BMI) significantly affected the community structure of the gut microbiota in *V. sinensis* (Age: *p*_adj_ = 0.006; BMI: *p*_adj_ = 0.024), while the environmental temperature did not significantly influence the gut microbiota (*p*_adj_ > 0.05). Age had the greatest effect, accounting for 12.1% of the community variation while BMI accounting for 9.5% (Fig. 4; Table 2).

**Table 2:** The results of RDA analysis. Effects of age (Age), environmental temperature (Temp), and body mass index (BMI) on the intestinal microbial community of Asian particolored bats were compared via RDA analysis.

Factor	Explains %	Pseudo-*F*		*P* (adj)
Age	12.1	5.9		0.006
BMI	9.5	4.5		0.024
Temp	2.8	1.2		0.792

## Discussion

Research has become increasingly focused on the vertebrate gut microbiota because the microbiota is closely related to health, growth and development. For organisms, the gut microbiota is indispensable and is even referred to as the body’s “second genome” (Bäckhed et al., 2007; O’Hara & Shanahan, 2006). However, bats occupy a special niche as they are nocturnal and primarily in the sky, leading to less research and attention to their gut microbiota. In this study, we characterized the gut microbial compositions of wild Asian particolored bats at various stages of development and the possible influencing factors on the changes in their gut microbial communities. Our results showed that the gut microbiota in *V. sinensis* was relatively stable at the phylum level during development, with Firmicutes and Proteobacteria comprising most of the community, which is similar to the study on the gut microbiota in adult *Hipposideros armiger* and *V. sinensis* (Xiao et al., 2019b). In addition, studies on the greater horseshoe bat showed that the main gut microbiota was comprised of Proteobacteria and Firmicutes (Xiao et al., 2019a, 2019b). In contrast, studies of the gut microbiota in other mammals have suggested that at the phylum level, the gut microbiota is dominated by Firmicutes and Bacteroidetes, which are associated with fat accumulation (Ley et al., 2008; Turnbaugh & Gordon, 2009).

The gut microbiota in the Asian particolored bats changed during the bats’ development. The alpha diversity increased in the early stages of ontogenesis (from day 1 to week 4). However, the increased diversity gradually leveled off and decreased significantly in the later stages (weeks 5 and 6). Beta diversity also showed that the microbiota from weeks 5 and 6 differed from those from week 1 to week 4. Teyssier et al. (2018) showed that the gut microbiota changed during early ontogenesis in great tits, with a significant decrease in diversity especially between 8 and 15 days. However, studies on *M. myotis* showed different results. In the developmental stage of 0–4 years, no significant change was detected in the gut microbiota (annual sampling), indicating that *M. myotis* may have a relatively stable, unchanging microbiota that play roles in extending a healthy life with the advancement of age and suggesting a potential link between the microbiota and sustained powered flight (Hughes et al., 2018). Studies on ontogeny of wild sparrows also revealed no significant changes in the gut microbiota composition during the first 12 days of early life (chick period) (Kohl et al., 2019).

A possible reason for the significant decrease in diversity in the gut microbiota in weeks 5 and 6 was the change in the Asian particolored bats’ diets during development. During field investigation, bats that were around 4 weeks old flew clumsily after being released back into the wild after sampling; Jin et al. (2012) also reported this finding. This flying behavior suggests that the bats may have been weaned and might hunt insects after week 4. Thus, the bats’ diet likely transitioned from breast milk to insects during that period. Some studies reported the weaning period and first flight time of other bat species, which are similar to our results. For example, the first flight and weaning period were between 25 and 30 days for *Plecotus auratus* (McLean, Annals & History, 2000), and the first flight time and weaning period were at 21 days and 29 days for *Pipistrellus subflavus*, respectively (Hoying & Kunz, 1998). In addition, community composition analysis showed that the alpha diversity of the gut microbiota gradually increased and became more abundant in week 4, then decreased in weeks 5 and 6, possibly indicating that the gut microbiota abundance in *V. sinensis* gradually decreased after weaning. After week 4, the relative abundances of *Brevibacterium* and *Brachybacterium* increased, while the *Enterobacter* abundance decreased. Some studies have shown that *Brevibacterium* plays a regulatory role in energy and carbohydrate metabolism (Ruklisha et al., 1981), while *Enterobacter* synthesizes fatty acids to meet the needs for growth and development (Hoffmann et al., 2008). Frese et al. (2015) obtained similar results, in which the diversities and community structures of the gut microbiota in pigs differed significantly before and after a change in diet. After introducing plant-based diets, the diversity of the porcine gut microbiota was significantly reduced, then gradually stabilized as the feeding habits became fixed (Frese et al., 2015). Our functional analysis showed that all functions of the gut microbiota had no significant difference before and after the predicted dietary transition at the level 2 and level 3 KEGG orthology groups. This is different from the gut microbiota function in pigs (Captivity) which is different before and after changes in diet (Frese et al., 2015). For *V. sinensis*, their diet changed from breast milk to insects. During this ontogenetic development process, the components of their food might be different, even if the main components of breast milk and insects are proteins (Bell, 1990; Jenness, 1988). While no useful information of predicted functional profiles were obtained in this study, which might be due to qualitative and quantitative differences between our data and the existing databases used by PICRUSt (Gajardo et al., 2016; Sullam et al., 2015). In our further study, more bats’ fecal samples should be collected and analyzed to improve the accuracy and predict microbial functionality regarding the dietary changes of *V. sinensis*.

Additionally, the alpha diversity of the Asian particolored bats on day 1 was higher than that in weeks 1 and 2 (Fig. 1). The reason for this inconsistency may be because our sampled individuals were not newly born and had been exposed to the environment, resulting in colonization via the environment or other sources, with high microbial diversity. In the early stages of life, the gut microbiota mainly reflects environmental conditions and the gut microbiota species that are conducive to the host’s growth are selectively increased over time (Burns et al., 2016; Trosvik, Stenseth & Rudi, 2010). Therefore, more samples should be included in subsequent studies to further analyze the reasons for the changes.

Many factors influence the community composition of the gut microbiota. Our study showed that age and BMI more significantly were associated with changes in the gut microbiota in bats than did the environmental temperature. As the Asian particolored bats age, their guts gradually mature, leading to inevitable dietary changes, which thus change the gut microbiota. Studies on gut microbiota changes in wild sparrows also showed that age was significantly correlated with gut microbiota changes (Kohl et al., 2019). In addition, during the development of Asian particolored bats, body weight and forearm length followed a linear growth pattern in the first 28 days of age, and body weight and forearm length were highly correlated with age. This suggests that the BMI was related to age. Therefore, the BMI accordingly affects the changes in the gut microbiota. Furthermore, sex may also be an important factor that influences changes in the microbiota. Phillips et al. (2012) showed that the sex of bats in the nonreproductive state did not significantly influence the gut microbiota. Thus, our study did not examine the influence of sex on the gut microbiota of Asian particolored bats.

## Conclusions

We analyzed the gut microbiota changes in Asian particolored bats and found that the gut microbiota changed during the bats’ development. Changes in the alpha and beta diversity during week 4 suggest a probable feeding transition, highlighting the importance of diet on the gut microbiota during *V. sinensis* development.

## Supplemental Information

10.7717/peerj.9003/supp-1 Supplemental Information 1 Sample information.

## Additional Information and Declarations

### Field Study Permissions

The following information was supplied relating to field study approvals (i.e., approving body and any reference numbers): The land in the Acheng district is publicly owned. All the field work has been certificated by College of Life Science, Jilin Agricultural University in China.

### DNA Deposition

The following information was supplied regarding the deposition of DNA sequences: The 16s amplicon sequences are available at GenBank: SRR9695884 to SRR9695940.

### Data Availability

The following information was supplied regarding data availability: Data is available at NCBI BioProject PRJNA554966.
