# Supplementary material for: Changes in the gut microbiota during Asian particolored bat (Vespertilio sinensis) development
Source: PeerJ. 2020 May 12;8:e9003. doi: 10.7717/peerj.9003 (PMC7227643; doi:10.7717/peerj.9003)
Supplement: Supplemental Information 1 [file peerj-08-9003-s001.doc]

**Table S1. Sample information.**

| **Sample ID** | **Body**  **temperature** | | **Environment temperature** | **Forearm length** | **Weight** | **Length of total gap** | **Date** |
| --- | --- | --- | --- | --- | --- | --- | --- |
| 1 | 36.1 | 26.6 | | 15.91 | 2.68 | NA | 2018-06-24 |
| 2 | 35 | 26.6 | | 15.34 | 2.67 | NA | 2018-06-24 |
| 3 | 32 | 26.6 | | 15.46 | 3.05 | NA | 2018-06-24 |
| 4 | 31.2 | 26.6 | | 16.03 | 3.06 | NA | 2018-06-24 |
| 5 | 34.1 | 26.8 | | 21.81 | 5.24 | NA | 2018-07-01 |
| 6 | 36 | 26.8 | | 21.74 | 5.89 | NA | 2018-07-01 |
| 7 | 32.3 | 26.8 | | 22.40 | 5.01 | NA | 2018-07-01 |
| 8 | 32 | 26.8 | | 24.49 | 6.54 | NA | 2018-07-01 |
| 9 | 33.5 | 26.8 | | 24.38 | 6.31 | NA | 2018-07-01 |
| 10 | 34.8 | 26.8 | | 24.96 | 6.46 | NA | 2018-07-01 |
| 11 | 33.5 | 20.1 | | 30.01 | 8.56 | NA | 2018-07-08 |
| 12 | 32.8 | 20.1 | | 29.55 | 8.93 | NA | 2018-07-08 |
| 13 | 34.7 | 20.1 | | 29.98 | 8.52 | NA | 2018-07-08 |
| 14 | 34.8 | 20.1 | | 30.13 | 7.82 | NA | 2018-07-08 |
| 15 | 32.5 | 20.1 | | 31.47 | 8.54 | NA | 2018-07-08 |
| 16 | 35.1 | 20.1 | | 30.69 | 8.79 | NA | 2018-07-08 |
| 17 | 33.9 | 20.1 | | 32.18 | 10.54 | NA | 2018-07-08 |
| 18 | 35.8 | 27.1 | | 38.09 | 10.04 | NA | 2018-07-15 |
| 19 | 36 | 27.1 | | 37.01 | 10.73 | NA | 2018-07-15 |
| 20 | 32.9 | 27.1 | | 39.1 | 9.56 | NA | 2018-07-15 |
| 21 | 34.1 | 27.1 | | 38.16 | 10.13 | NA | 2018-07-15 |
| 22 | 34.1 | 27.1 | | 39.2 | 11.42 | NA | 2018-07-15 |
| 23 | 32.3 | 27.1 | | 39.45 | 11.28 | NA | 2018-07-15 |
| 24 | 34.8 | 27.1 | | 39.78 | 11.04 | NA | 2018-07-15 |
| 25 | 35 | 31.1 | | 47.83 | 14.88 | NA | 2018-07-22 |
| 26 | 34.3 | 31.1 | | 47.15 | 14.91 | NA | 2018-07-22 |
| 27 | 34 | 31.1 | | 47.67 | 14.03 | NA | 2018-07-22 |
| 28 | 34.2 | 31.1 | | 48.06 | 14.73 | NA | 2018-07-22 |
| 29 | 36.1 | 31.1 | | 47.7 | 16.95 | NA | 2018-07-22 |
| 30 | 35.6 | 31.1 | | 47.48 | 13.64 | NA | 2018-07-22 |
| 31 | 33.9 | 31.1 | | 47.32 | 12.28 | NA | 2018-07-22 |
| 32 | 35.9 | 31.1 | | 48.61 | 15.37 | 2.45 | 2018-07-22 |
| 33 | 34.3 | 31.7 | | 51.5 | 16.63 | 2.36 | 2018-07-29 |
| 34 | 32.3 | 31.7 | | 50.12 | 15.51 | 2.27 | 2018-07-29 |
| 35 | 34.6 | 31.7 | | 51.45 | 16.8 | 1.92 | 2018-07-29 |
| 36 | 33.7 | 31.7 | | 49.49 | 15.22 | 1.86 | 2018-07-29 |
| 37 | 33.3 | 31.7 | | 50.21 | 14.71 | 1.85 | 2018-07-29 |
| 38 | 33.8 | 31.7 | | 50.35 | 16.62 | 1.21 | 2018-07-29 |
| 39 | 33 | 21.8 | | 49.49 | 19.11 | 1.21 | 2018-08-05 |
| 40 | 32.7 | 21.8 | | 49.56 | 18.43 | 1.19 | 2018-08-05 |
| 41 | 33.5 | 21.8 | | 49.78 | 18.51 | 1.18 | 2018-08-05 |
| 42 | 35.8 | 21.8 | | 50.75 | 17.94 | 0.84 | 2018-08-05 |
| 43 | 36 | 21.8 | | 50.41 | 19.72 | 0.76 | 2018-08-05 |
| 44 | 35.4 | 21.8 | | 51.32 | 18.4 | 0.61 | 2018-08-05 |
| 45 | 33.3 | 21.8 | | 49.18 | 18.25 | 0.59 | 2018-08-05 |

NA : this age stage parameter is not available.
